# Supplementary material for: Metabolic signatures of ferritin and TDP-43 co-pathology provide a mechanistic basis for stratified therapeutic approaches in ALS
Source: bioRxiv. 2026 Mar 16:2026.03.13.711539. Preprint. [Version 1] doi: 10.64898/2026.03.13.711539 (PMC13015557; doi:10.64898/2026.03.13.711539)
Supplement: Supplement 2 [file NIHPP2026.03.13.711539v1-supplement-2.pdf]

bioRxiv preprint doi: https://doi.org/10.64898/2026.03.13.711539; this version posted March 16, 2026. The copyright holder for this preprint (which was not certified by peer review) is the author/funder, who has granted bioRxiv a license to display the preprint in perpetuity. It is made available under aCC-BY 4.0 International license.

# Supplementary Materials

**Supplementary figure 1.** Tuning the number of components in PLS-DA demonstrates that for all prediction distances assessed, a 2-component model was optimal. For each component, repeated cross-validation (10 × 3–fold CV) was used to evaluate the PLS-DA classification performance (OER and BER), for each type of prediction distance; `max.dist`, `centroids.dist` and `mahalanobis.dist`.

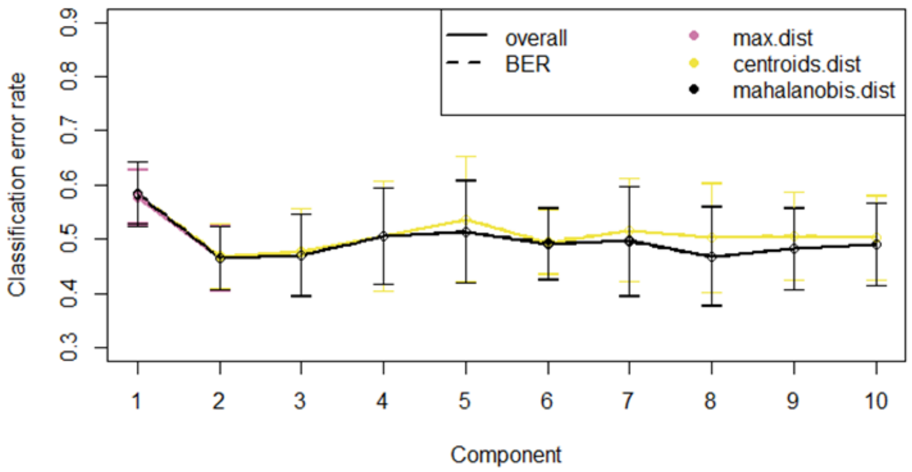


**Supplementary figure 2.** Three component PLSDA model separating TDP-43 and ferritin pathology groups with points labelled by disease status rather than pathology group. The identified altered metabolites explain variance in data dependent on pathology presence but lack explanation of separation between disease vs control.

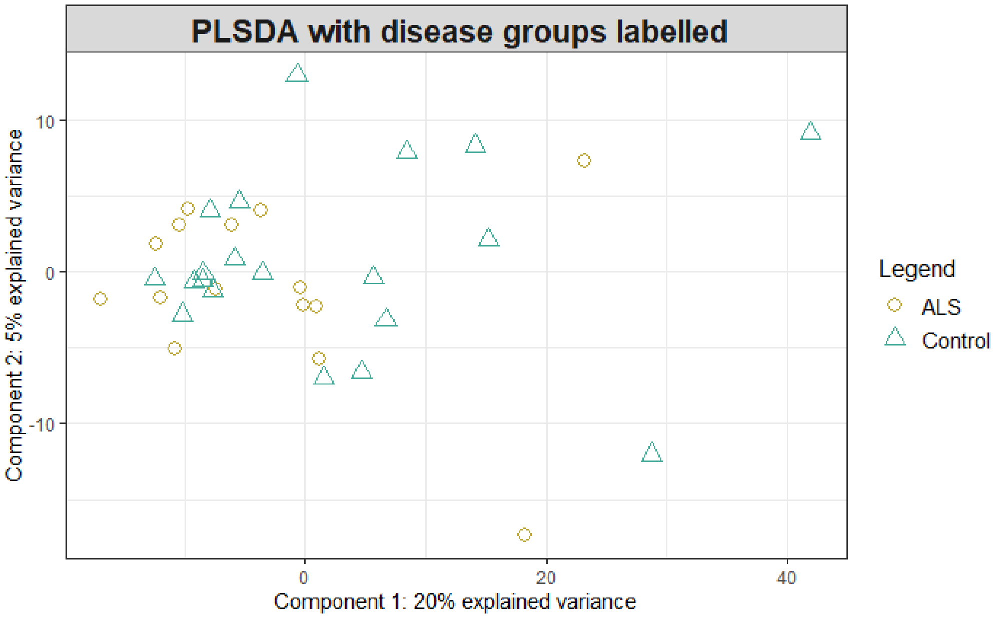

bioRxiv preprint doi: https://doi.org/10.64898/2026.03.13.711539; this version posted March 16, 2026. The copyright holder for this preprint (which was not certified by peer review) is the author/funder, who has granted bioRxiv a license to display the preprint in perpetuity. It is made available under aCC-BY 4.0 International license.

**Supplementary figure 3.** Boxplots to show relationships between metabolites with top 10 VIP scores and pathology status.

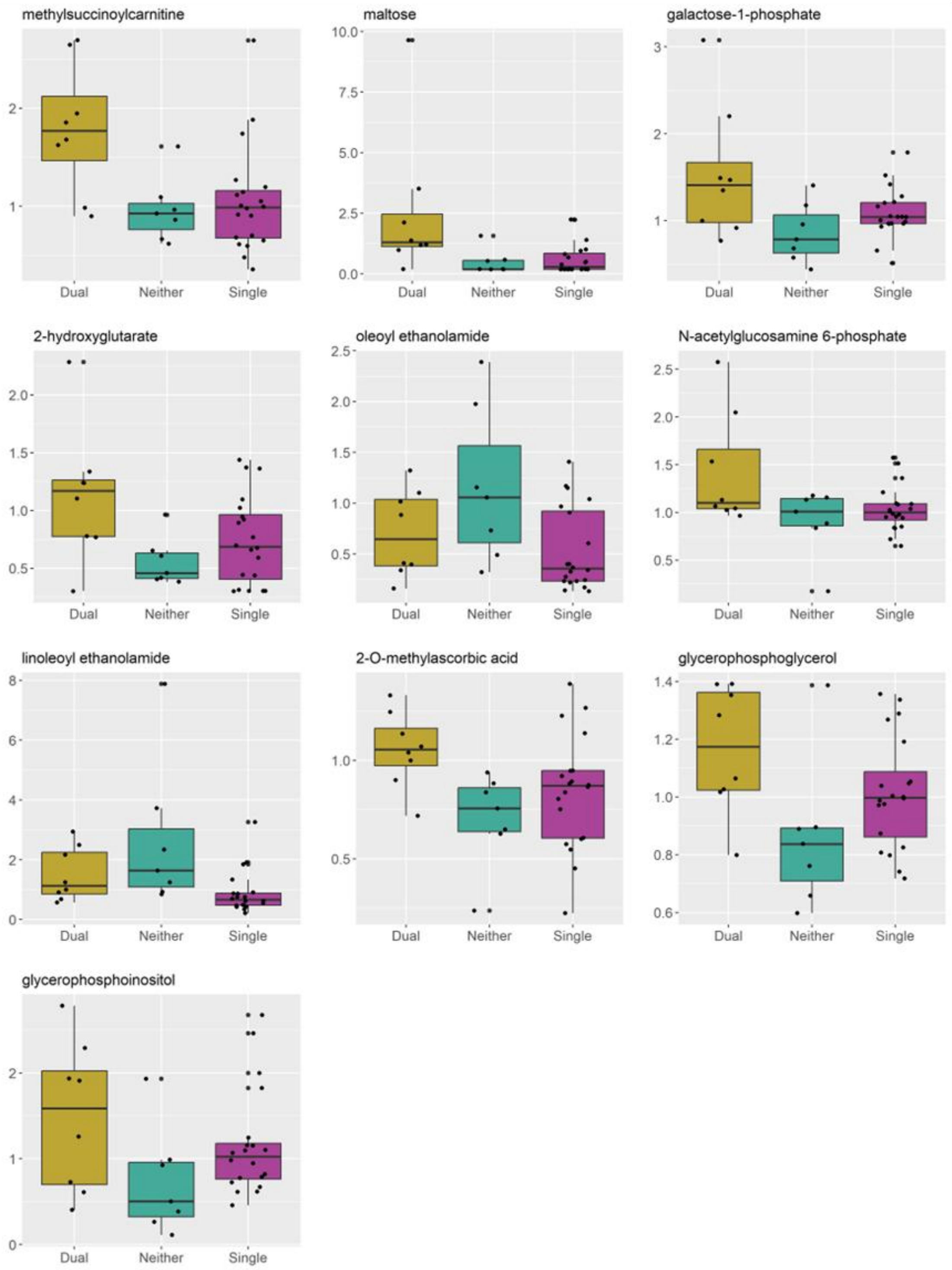

bioRxiv preprint doi: https://doi.org/10.64898/2026.03.13.711539; this version posted March 16, 2026. The copyright holder for this preprint (which was not certified by peer review) is the author/funder, who has granted bioRxiv a license to display the preprint in perpetuity. It is made available under aCC-BY 4.0 International license.

**Supplementary figure 4.** There is no significant correlation between TDP-43 and GPX4 when assessing superpixel burden score (Spearman; R=-0.16; p=0.4).

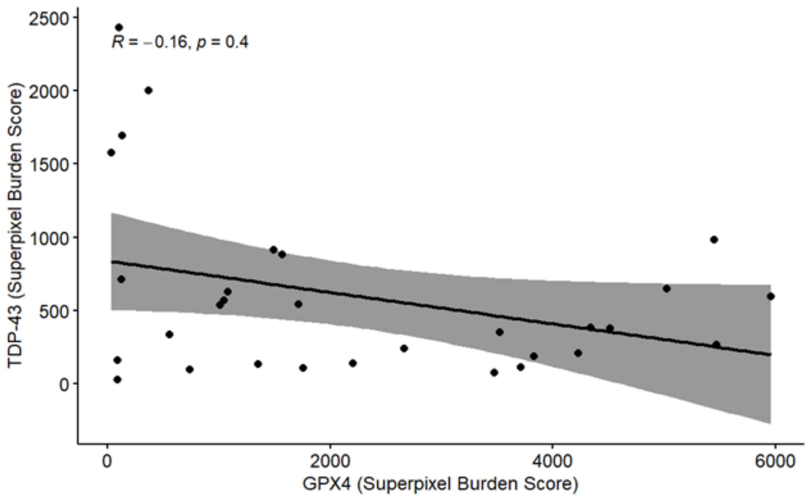


706
